# Work stress and risk of death in men and women with and without cardiometabolic disease: a multicohort study

**DOI:** 10.1016/S2213-8587(18)30140-2

**Published:** 2018-09

**Authors:** Mika Kivimäki, Jaana Pentti, Jane E Ferrie, G David Batty, Solja T Nyberg, Markus Jokela, Marianna Virtanen, Lars Alfredsson, Nico Dragano, Eleonor I Fransson, Marcel Goldberg, Anders Knutsson, Markku Koskenvuo, Aki Koskinen, Anne Kouvonen, Ritva Luukkonen, Tuula Oksanen, Reiner Rugulies, Johannes Siegrist, Archana Singh-Manoux, Sakari Suominen, Töres Theorell, Ari Väänänen, Jussi Vahtera, Peter J M Westerholm, Hugo Westerlund, Marie Zins, Timo Strandberg, Andrew Steptoe, John Deanfield

**Affiliations:** a Clinicum, Faculty of Medicine, and Helsinki Institute of Life Science, University of Helsinki, Helsinki, Finland; b Institute of Behavioural Sciences, University of Helsinki, Helsinki, Finland; c Department of Public Health, University of Helsinki, Helsinki, Finland; d Faculty of Social Sciences, University of Helsinki, Helsinki, Finland; e Department of Epidemiology and Public Health, University College London, London, UK; f National Centre for Cardiovascular Prevention and Outcomes, University College London, London, UK; g Department of Public Health, University of Turku, Turku, Finland; h School of Social and Community Medicine, University of Bristol, Bristol, UK; i Institute of Public Health and Caring Sciences, University of Uppsala, Uppsala, Sweden; j Centre for Occupational and Environmental Medicine, Stockholm County Council, Stockholm, Sweden; k Institute of Environmental Medicine, Karolinska Institutet, Stockholm, Sweden; l Institute for Medical Sociology, Medical Faculty, University of Düsseldorf, Düsseldorf, Germany; m School of Health and Welfare, Jönköping University, Jönköping, Sweden; n Stress Research Institute, Stockholm University, Stockholm, Sweden; o Inserm UMS 011, Population-Based Epidemiological Cohorts Unit, Villejuif, France; p Versailles St-Quentin University, UMS 011, Villejuif, France; q Department of Health Sciences, Mid Sweden University, Sundsvall, Sweden; r Finnish Institute of Occupational Health, Helsinki, Finland; s Division of Health Psychology, SWPS University of Social Sciences and Humanities in Wroclaw, Wroclaw, Poland; t Administrative Data Research Centre Northern Ireland, Centre for Public Health, Queen's University Belfast, Belfast, UK; u National Research Centre for the Working Environment, Copenhagen, Denmark; v Department of Public Health and Department of Psychology, University of Copenhagen, Copenhagen, Denmark; w Inserm UMR 1018, Centre for Research in Epidemiology and Population Health, Villejuif, France; x Folkhälsan Research Center, Helsinki, Finland; y School of Health and Education, University of Skövde, Skövde, Sweden; z School of Social Policy, Sociology and Social Research, University of Kent, Canterbury, UK; aa Turku University Hospital, Turku, Finland; ab Department of Medical Sciences, Uppsala University, Uppsala, Sweden; ac Department of Internal Medicine, Helsinki University Hospital, Helsinki, Finland; ad Center for Life Course Health Research, University of Oulu, Oulu, Finland

## Abstract

**Background:**

Although some cardiovascular disease prevention guidelines suggest a need to manage work stress in patients with established cardiometabolic disease, the evidence base for this recommendation is weak. We sought to clarify the status of stress as a risk factor in cardiometabolic disease by investigating the associations between work stress and mortality in men and women with and without pre-existing cardiometabolic disease.

**Methods:**

In this multicohort study, we used data from seven cohort studies in the IPD-Work consortium, initiated between 1985 and 2002 in Finland, France, Sweden, and the UK, to examine the association between work stress and mortality. Work stress was denoted as job strain or effort–reward imbalance at work. We extracted individual-level data on prevalent cardiometabolic diseases (coronary heart disease, stroke, or diabetes [without differentiation by diabetes type]) at baseline. Work stressors, socioeconomic status, and conventional and lifestyle risk factors (systolic and diastolic blood pressure, total cholesterol, smoking status, BMI, physical activity, and alcohol consumption) were also assessed at baseline. Mortality data, including date and cause of death, were obtained from national death registries. We used Cox proportional hazards regression to study the associations of work stressors with mortality in men and women with and without cardiometabolic disease.

**Results:**

We identified 102 633 individuals with 1 423 753 person-years at risk (mean follow-up 13·9 years [SD 3·9]), of whom 3441 had prevalent cardiometabolic disease at baseline and 3841 died during follow-up. In men with cardiometabolic disease, age-standardised mortality rates were substantially higher in people with job strain (149·8 per 10 000 person-years) than in those without (97·7 per 10 000 person-years; mortality difference 52·1 per 10 000 person-years; multivariable-adjusted hazard ratio [HR] 1·68, 95% CI 1·19–2·35). This mortality difference for job strain was almost as great as that for current smoking versus former smoking (78·1 per 10 000 person-years) and greater than those due to hypertension, high total cholesterol concentration, obesity, physical inactivity, and high alcohol consumption relative to the corresponding lower risk groups (mortality difference 5·9–44·0 per 10 000 person-years). Excess mortality associated with job strain was also noted in men with cardiometabolic disease who had achieved treatment targets, including groups with a healthy lifestyle (HR 2·01, 95% CI 1·18–3·43) and those with normal blood pressure and no dyslipidaemia (6·17, 1·74–21·9). In all women and in men without cardiometabolic disease, relative risk estimates for the work stress–mortality association were not significant, apart from effort–reward imbalance in men without cardiometabolic disease (mortality difference 6·6 per 10 000 person-years; multivariable-adjusted HR 1·22, 1·06–1·41).

**Interpretation:**

In men with cardiometabolic disease, the contribution of job strain to risk of death was clinically significant and independent of conventional risk factors and their treatment, and measured lifestyle factors. Standard care targeting conventional risk factors is therefore unlikely to mitigate the mortality risk associated with job strain in this population.

**Funding:**

NordForsk, UK Medical Research Council, and Academy of Finland.

## Introduction

Meta-analyses of prospective cohort studies have shown that psychosocial stress might increase the risk of cardiovascular disease and diabetes.^1^, ^2^, ^3^, ^4^ The underlying pathophysiological mechanisms include disturbed sympathetic-parasympathetic balance and dysregulation of the hypothalamic–pituitary–adrenal axis, which can accelerate the development of metabolic syndrome and lead to left-ventricular dysfunction, dysrhythmia, and proinflammatory and procoagulant responses.^5^, ^6^ Stress has also been linked to worsening health-related lifestyle factors, such as physical inactivity and increased alcohol consumption, and, in people with existing illness, suboptimal treatment adherence.^6^

Although prevention guidelines for cardiovascular disease do not prioritise the management of stress in the general population,^7^, ^8^, ^9^ some guidelines recommend stress management for individuals with established cardiovascular disease or major cardiovascular risk factors, such as diabetes.^7^ The rationale for these recommendations is that people with cardiometabolic disease have many more adverse health events than do the general population—therefore, assuming that the relative risk associated with stress is the same for all people exposed, a greater number of adverse events will be prevented by targeting those already at high risk. However, the evidence base for this recommendation is weak, relying on studies of disease incidence^1^, ^2^, ^3^, ^4^, ^10^, ^11^ with very few large-scale studies of mortality^11^, ^12^, ^13^, ^14^, ^15^ and stress in patients with cardiometabolic disease.^13^, ^14^, ^15^, ^16^, ^17^ Importantly, it is unknown whether the excess risk associated with stress at work and private life can be mitigated by controlling conventional risk factors (eg, blood pressure and cholesterol concentration) and improving lifestyle (eg, physical activity and weight control).

**Research in context**

**Evidence before this study**

Work stressors, such as job strain and effort–reward imbalance at work, are common sources of stress in adulthood. Work stressors have been examined as risk factors for cardiometabolic disease, such as coronary heart disease, stroke, and diabetes, but few studies are available on their role as prognostic factors for these diseases. We searched PubMed and Embase databases from inception up to Feb 1, 2018 using the search terms: “work stress”, “job stress”, “job strain”, “effort–reward imbalance”, and “mortality”, without language restrictions. We identified no large-scale studies comparing the association between work stressors and mortality in people with and without cardiometabolic disease.

**Added value of this study**

We pooled individual-participant data from seven European cohort studies, including a total of 102 633 men and women. Job strain was associated with substantial relative and absolute increases in mortality risk in men with cardiometabolic disease. The mortality difference between groups with and without job strain was clinically significant and independent of socioeconomic status and several conventional and lifestyle risk factors, including hypertension and dyslipidaemia and their pharmacological treatments, obesity, smoking, physical inactivity, and high alcohol consumption. In absolute terms, the difference in age-standardised mortality was greater for current smoking versus not smoking than for with versus without job strain, but, job strain was associated with a greater mortality difference than were high cholesterol, obesity, high alcohol consumption, and physical inactivity. In women and participants without cardiometabolic disease, the work stress–mortality associations were small or absent, both in relative and absolute terms.

**Implications of all the available evidence**

The finding that job strain increases mortality risk, even in subgroups of men with cardiometabolic disease but a favourable cardiometabolic risk profile, suggests that standard care targeting conventional risk factors is unlikely to mitigate the mortality risk associated with job strain. Subsequent research should employ intervention designs to establish whether systematic screening and management of work stressors such as job strain would contribute to improved health outcomes in men with coronary heart disease, stroke, or diabetes.

The Individual-Participant-Data Meta-analysis in Working Populations (IPD-Work) consortium is the largest multicohort research collaboration on work stress and clinically verified cardiovascular disease and diabetes.^1^, ^3^, ^10^ In this study, we sought to clarify the status of stress as a risk factor in cardiometabolic disease by investigating the associations of two common work stressors, job strain and effort–reward imbalance, with mortality in individuals with pre-existing diabetes or coronary heart disease or a history of stroke. For comparison, we examined the stress–mortality association in individuals without these diseases. To investigate whether management of conventional and lifestyle risk factors is likely to eliminate any excess risk associated with work stress, we also assessed the stress–mortality relation among patients with cardiometabolic disease who otherwise had low risk factor levels (ie, were normotensive, non-obese, physically active, had normal blood cholesterol concentrations, and were not smokers or heavy drinkers). If stress was associated with excess mortality, even in subgroups of low-risk patients, then better stress management might be an improvement on standard care.

## Methods

### Study population

Established in 2008, the objective of the IPD-Work Consortium^1^, ^10^ is to provide a large-scale harmonised database for the longitudinal estimation of associations between predefined psychosocial working conditions and chronic disease outcomes. The participating studies comply with the Declaration of Helsinki and were approved by local ethics review boards. Informed consent was obtained from all participants.

Of the 12 original studies in the IPD-Work Consortium,^1^ seven independent cohort studies, initiated between 1985 and 2002 in Finland (FPS, HeSSup, Still Working), France (GAZEL), Sweden (WOLF S, WOLF N) and the UK (Whitehall II), had data relevant to the present research. From each cohort study, eligible participants were those who were employed at the time of the baseline assessment, had data for age, sex, job strain, effort–reward imbalance at work, and prevalent cardiovascular disease and diabetes, and were being followed up for mortality. Data were anonymised and available at the individual level. Details of the studies included in the present multicohort analysis are summarised in the appendix.

### Clinical characteristics

Baseline characteristics recorded were age, sex, and harmonised measures of smoking (never smoker, ex-smoker, or current smoker), alcohol consumption (non-drinkers, moderate drinkers [1–14 drinks per week for women and 1–21 drinks per week for men], and heavy drinkers [>14 drinks per week for women and >21 drinks per week for men]), leisure-time physical activity (none or very little, moderate, or vigorous physical activity or exercise), BMI (<18·5 kg/m^2^ [underweight], 18·5–24·9 kg/m^2^ [normal weight], 25–29·9 kg/m^2^ [overweight], or ≥30 kg/m^2^ [obese]), and socioeconomic status (high, intermediate, or low, defined on the basis of an occupational title or, in the HeSSup study, a participant's highest educational qualification).^1^ In three studies (Whitehall II, WOLF S, and WOLF N), assessments of systolic and diastolic blood pressure and total cholesterol concentration were also available.^1^ In four studies (FPS, HeSSup, WOLF S, and WOLF N), it was possible to assess prescriptions in participants with diabetes, coronary heart disease, or stroke by linkage to prescription registers during the baseline year. Prescriptions for antidiabetes (Anatomical Therapeutic Chemical [ATC] code A10), antihypertensive (ATC C02, C03, C07–C09), lipid-lowering (ATC C10AA), and anticoagulation (ATC B01) medications were considered to indicate adherence.

### Work stress

Analyses were based on two indicators of work stress: job demand–control (ie, job strain) and effort–reward imbalance at work. Reports from the IPD-Work consortium are based on predefined, harmonised, and validated definitions of work stress. The psychometric properties of these data were published before the extraction of outcome data.^18^, ^19^ Job strain, referring to a combination of high demands and low control at work, was measured with sets of questions from the validated Job Content Questionnaire and Demand-Control Questionnaire, which were included in the baseline self-report questionnaire of all of seven studies.^18^ Using both questionnaires, we defined high job demands as having a job-demand score that was greater than the study-specific median score; similarly, we defined low job control as having a job control score that was lower than the study-specific median score. The Pearson correlations between the harmonised scales used in this study and complete versions of the Job Content Questionnaire and Demand Control Questionnaire all had *r* greater than 0·9, apart from one study in which *r* was 0·8.^18^ In the present analyses, the exposure was defined as job strain versus no job strain according to the job strain model.^1^

The Effort–Reward Imbalance at Work questionnaire at baseline included items on work demands and efforts (the effort items) and monetary and non-monetary rewards at work (the reward items). Different questionnaire versions were harmonised and validated across the constituent studies before the mortality analyses.^19^ Pearson correlation coefficients between the harmonised scales used in this study and complete versions of the Effort–Reward Imbalance questionnaire were high: *r* was greater than 0·9 for the effort scales and greater than 0·8 for the reward scales.^19^ For each participant, mean response scores were calculated separately for the effort and reward items. We constructed a ratio of the two scores to quantify the degree of mismatch between effort and rewards. The effort–reward ratio was dichotomised at a cutoff point of 1 with a ratio greater than 1 indicating effort–reward imbalance and a ratio of 1 or lower indicating no effort–reward imbalance at work.^19^

To examine the combined effects of job strain and effort–reward imbalance, we constructed a three-level exposure variable, where 0 represented no job strain or effort–reward imbalance, 1 represented either job strain or effort–reward imbalance (not both), and 2 represented both job strain and effort–reward imbalance.^2^ More details of the work stress measurements are provided in the appendix.

### Baseline cardiometabolic disease

Baseline (existing) cardiometabolic diseases included common causes of death: coronary heart disease, stroke, and diabetes (without distinguishing between types of diabetes). Coronary heart disease was ascertained from national hospital admission records and discharge registries (participants were linked to these registers with individual identification numbers) and denoted with version 10 of the International Classification of Diseases (ICD; codes I21–I22 or the corresponding ICD-9 or ICD-8 codes),^1^ or clinical examination with the MONICA definition (Whitehall II).^20^ Agreement between the national hospital admission records and clinical examinations for coronary heart disease has been shown to be high (sensitivity 70% and specificity >95%, with clinical examination used as the gold standard ascertainment method).^21^ We identified history of stroke using self-reported doctor-diagnosed events, event tracing, and linkage to national hospital admission records (ICD-10 codes I60, I61, I63, I64).^10^ Prevalent diabetes was defined with information from any of the following data sources: hospital admission records with ICD-10 diagnoses (E10, E11; all studies apart from Whitehall II), antidiabetes drug reimbursements (only FPS, Still Working, and HeSSup),^3^ or 2 h oral glucose tolerance test (WHO criteria) complemented by self-report of diabetes diagnosis and medication (Whitehall II).^22^

### Mortality follow-up

Mortality data, including date and cause of death, were obtained from national death registries. In each study, participants were linked to mortality records using their unique identification numbers. Because these records do not include date of death for people who emigrate and die abroad, such participants were flagged as emigrants and censored at the date of emigration.

### Statistical analysis

Means and SDs were calculated according to cardiometabolic disease status at baseline (prevalent coronary heart disease, stroke, or diabetes *vs* none of these diseases). Each participant was followed up from the date of the assessment of their work stressors and prevalent cardiometabolic disease to the earliest event out of death, loss to follow-up, or end of follow-up (maximum 20 years). We computed time to death to obtain age-adjusted incidence rates per 10 000 person-years in men and women in the pooled dataset. We used Cox proportional hazards regression to study the associations of work stressors (job strain and effort–reward imbalance at work) with mortality in men and women with and without cardiometabolic disease. Bonferroni correction was used to compensate for multiple testing (a total of eight tests from two stressors, two cardiometabolic disease statuses, and two sexes). Minimally adjusted models included age and study as covariates. Multivariable models were also adjusted for socioeconomic status, BMI category, smoking status, alcohol consumption, physical activity and, in a subgroup analysis of cohort studies with relevant data, blood pressure and total cholesterol concentration. Interactions with age were tested by grouping participants by age (<45 years, 45–54 years, and >55 years). Heterogeneity in study-specific estimates was examined by repeating the main analyses with a two-step procedure, with separate analyses in each cohort study and then pooling of the study-specific hazard ratios by use of random-effects meta-analysis.

Robustness of the association between work stressors and mortality in subgroups was tested in analyses stratified by number of lifestyle risk factors (zero, one, or two or more from current smoking, physical inactivity, obesity, and high alcohol consumption). To examine whether any effect of job strain is present in individuals with cardiometabolic disease but an otherwise low-risk profile, we assessed the association between work stressors and mortality in subgroups of participants who had met treatment targets—ie, they had adhered to pharmacotherapy, had normal blood pressure (systolic and diastolic blood pressure <140/90 mm Hg), and normal fasting total cholesterol concentration (<6·2 mmol/L and, in sensitivity analysis, <5·0 mmol/L). To minimise residual confounding, systolic blood pressure and total cholesterol concentration, treated as continuous variables, were added to the model as covariates.

In further analyses, we examined the association of exposure to neither, either, or both of the work stressors with mortality and the associations of obesity, current smoking, high alcohol consumption, and physical inactivity with mortality.

All analyses were done with SAS statistical software version 9.4. Statistical significance was inferred at a two-sided p value less than 0·05.

### Role of the funding source

The funders of the study had no role in study design, data collection, data analysis, data interpretation, or writing of the report. MKi and JP had full access to all the data in the study and MKi and JD had final responsibility for the decision to submit for publication.

## Results

**Figure 1:**
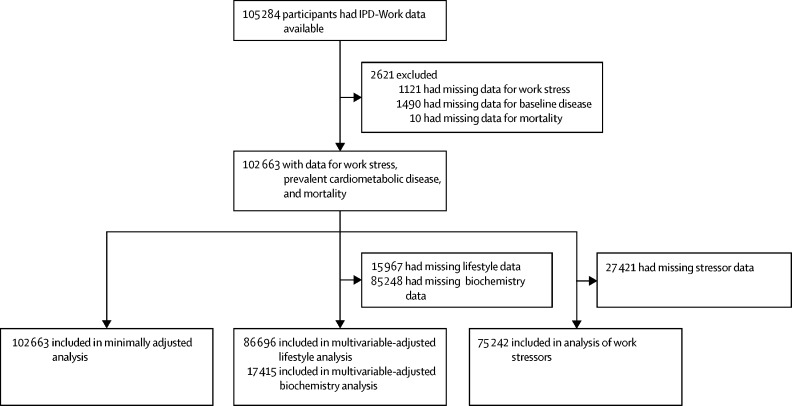
Sample selection

105 284 people were recruited into the seven studies between 1985 and 2002. Of the eligible population, 102 663 participants had data on prevalent cardiometabolic disease, at least one of the work stressors (job strain or effort–reward imbalance), and mortality, and were therefore included in this study (figure 1). Characteristics were similar between the eligible and included populations in terms of the proportion of men (43·5% in eligible *vs* 43·4% in enrolled participants), mean age (44·0 years *vs* 43·9 years), and proportion of participants of low-socioeconomic status (26·2% *vs* 25·8%).

Mean follow-up for mortality was 13·9 years (SD 3·9). During 1 423 753 person-years at risk, we identified 3841 deaths, of which 397 were among the 3441 individuals with cardiometabolic disease at baseline. Of the 1975 men with cardiometabolic disease at baseline, 396 had a history of coronary heart disease, 214 had stroke, 1425 had diabetes, 54 had two of these disorders, and three had all three. Of the 1466 women with cardiometabolic disease at baseline, 73 had a history of coronary heart disease, 153 had stroke, 1266 had diabetes, 18 had two of these disorders, and four had all three (appendix).

**Figure 2:**
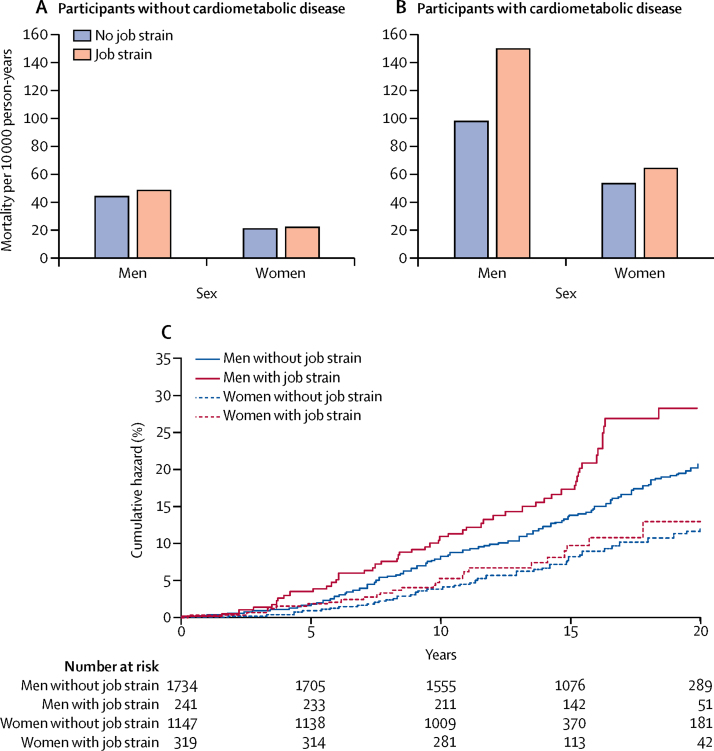
Job strain and age-adjusted mortality Job strain and mortality in participants without (A) and with cardiometabolic disease (B) at baseline, and cumulative hazard in participants with cardiometabolic disease at baseline (C).

In men without cardiometabolic disease at baseline, effort–reward imbalance was associated with an increased risk of mortality (mortality difference 6·6 per 10 000 person-years), and this association remained after multivariable adjustment and correction for multiple testing (table 1). There was no significant heterogeneity in the study-specific estimates (*I*^2^=0%, p=0·44; appendix) or interaction with age (p=0·10). In absolute terms, the difference in mortality between those with and without effort–reward imbalance was smaller than those related to conventional lifestyle factors, such as smoking, physical inactivity, and obesity (appendix). In women without cardiometabolic disease at baseline, effort–reward imbalance was not associated with mortality (table 1). Job strain alone (table 1 and figure 2A), or in combination with effort–reward imbalance (appendix), was not associated with mortality in men or women without cardiometabolic disease.

**Table 1:** Association between work stressors and total mortality in men and women, by baseline cardiometabolic disease

		**Participants without prevalent cardiometabolic disease (n=99 222)**						**Participants with prevalent cardiometabolic disease (total n=3441)**					
		Deaths/participants	Minimally adjusted*		Multivariable adjusted†			Deaths/participants	Minimally adjusted*		Multivariable adjusted†		
			HR (95% CI)	p value	HR (95% CI)	p value	p_corrected_‡		HR (95% CI)	p value	HR (95% CI)	p value	p_corrected_‡
**Men**													
Job strain													
	No	2049/37 287	1 (ref)	..	1 (ref)	..	..	256/1734	1 (ref)	..	1 (ref)	..	..
	Yes	296/5246	1·06 (0·94–1·20)	0·35	1·01 (0·86–1·19)	0·92	1·00	51/241	1·66 (1·23–2·25)	0·001	1·68 (1·19–2·35)	0·003	0·024
Effort–reward imbalance													
	No	755/19 675	1 (ref)	..	1 (ref)	..	..	133/1027	1 (ref)	..	1 (ref)	..	..
	Yes	340/7911	1·21 (1·05–1·39)	0·009	1·22 (1·06–1·41)	0·006	0·048	61/498	0·70 (0·51–0·97)	0·03	0·70 (0·50–0·98)	0·04	0·32
**Women**													
Job strain													
	No	972/46 242	1 (ref)	..	1 (ref)	..	..	77/1147	1 (ref)	..	1 (ref)	..	..
	Yes	247/10 447	1·05 (0·91–1·20)	0·52	0·96 (0·82–1·12)	0·59	1·00	26/319	1·21 (0·78–1·90)	0·40	1·11 (0·68–1·84)	0·67	1·00
Effort–reward imbalance													
	No	559/28 280	1 (ref)	..	1 (ref)	..	..	46/703	1 (ref)	..	1 (ref)	..	..
	Yes	284/16 611	0·93 (0·80–1·07)	0·32	0·91 (0·78–1·06)	0·23	1·00	32/537	1·01 (0·64–1·60)	0·96	0·99 (0·62–1·60)	0·98	1·00

HR=hazard ratio.

* Minimal adjustment includes age and study.

† Multivariable adjustment includes study, age, smoking status, physical activity, alcohol consumption, BMI, and socioeconomic status.

‡ p value corrected for multiple testing (Bonferroni correction).

In men with cardiometabolic disease at baseline, job strain was associated with an increased risk of mortality that remained after multivariable adjustment and correction for multiple testing (table 1). The age-adjusted mortality rate per 10 000 person-years was 149·8 in men with job strain and 97·7 in those without (risk difference 52·1 per 10 000; figure 2B). The excess mortality risk in men with cardiometabolic disease who reported job strain was apparent across the entire follow-up period, rather than becoming apparent only in the early or late phases of the follow-up (figure 2C), and was robust to adjustment for lifestyle risk factors (smoking status, alcohol consumption, physical activity, BMI, and socioeconomic status; table 1). There was no significant heterogeneity in study-specific estimates (*I*^2^=0%, p=0·96; appendix) or difference in the association between age groups (p_interaction_=0·62). After further adjustment for blood pressure and total cholesterol in the subgroup of participants with these data available, the HR for job strain compared with no job strain was 1·84 (95% CI 1·06–3·18; p=0·029; 94 deaths among 569 participants). In analyses of cause-specific mortality, job strain had a minimally adjusted (study and age) HR of 1·71 (95% CI 1·08–2·71; p=0·02) for risk of mortality from cardiovascular disease (appendix), but no robust associations were observed with cancer mortality or non-cardiovascular, non-cancer mortality. Effort–reward imbalance seemed to be associated with lower risk of death in men with previous cardiometabolic disease, but this association was lost after correction for multiple testing (table 1).

In women with cardiometabolic disease at baseline, the age-adjusted death rates per 10 000 were 64·0 for job strain and 53·2 for no job strain (difference 10·8 per 10 000; figure 2) and mortality was not significantly associated with job strain or effort–reward imbalance (table 1). Job strain in combination with effort–reward imbalance was not associated with mortality in men or women with cardiometabolic disease (appendix).

**Figure 3:**
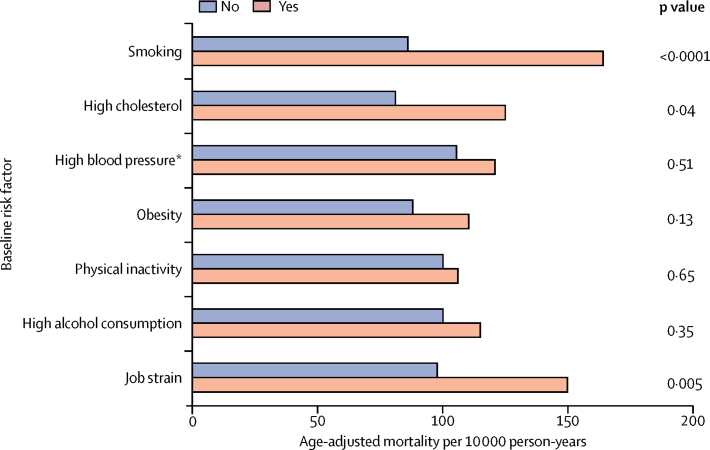
Mortality in men with cardiometabolic disease by job strain and lifestyle factors *Data available only from Whitehall II, WOLF-N, and WOLF-S.

**Figure 4:**
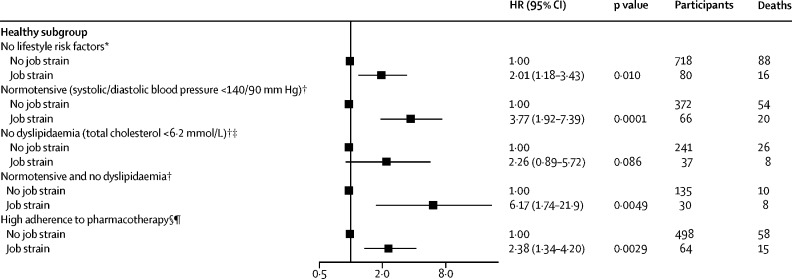
Job strain and mortality in men with cardiometabolic disease and a favourable risk profile HRs are adjusted for age and study. In analyses of normotensive and non-dyslipidaemic participants, HRs are also adjusted for systolic and diastolic blood pressure and total cholesterol. HR=hazard ratio. *Analysis included all studies. †Analysis included Whitehall II, WOLF-N, and WOLF-S. ‡For a subgroup of participants with total cholesterol <5·0 mmol/L, the corresponding hazard ratio is 4·82 (95% CI 1·15–20·2) for those with job strain (three deaths among nine participants) compared with those without job strain (five deaths among 71 participants). §Antidiabetes (ATC A10), antihypertensive (ATC C02, C03, C07-C09), lipid-lowering (ATC C10AA), and anticoagulation (ATC B01) medication. ¶Analysis includes FPS, HeSSup, WOLF-S, WOLF-N.

To examine the relative importance of job strain as a risk factor for mortality in men with cardiometabolic disease, we compared death rates associated with job strain with those associated with established risk factors. The mortality difference between men with and without job strain (52·1 per 10 000) was almost the same as that for current smokers versus never or former smokers (78·1 per 10 000), and higher than those for the presence of hypertension, high total cholesterol, obesity, physical inactivity, and high alcohol consumption (5·9–44·0 per 10 000; figure 3). Furthermore, job strain was associated with a two to six times higher risk of mortality in subgroups of men with cardiometabolic disease but favourable risk factor profiles, including participants who were not obese, physically inactive, smokers, or heavy drinkers (table 2) and normotensive participants, those with no dyslipidaemia, and those who adhered to antihypertensive, lipid-lowering, or anticoagulation treatments, according to prescription records (figure 4). Additional adjustments did not alter these findings (appendix).

**Table 2:** Association between job strain and mortality by number of lifestyle risk factors in men with cardiometabolic disease at baseline

	**Job strain**	**Participants**	**Deaths**	**HR (95% CI)**	**p value**
0 lifestyle risk factors	No	718	88	1 (ref)	..
0 lifestyle risk factors	Yes	80	16	2·01 (1·18–3·43)	0·010
1 lifestyle risk factor	No	626	94	1·39 (1·03–1·86)	0·029
1 lifestyle risk factor	Yes	95	19	2·09 (1·27–3·45)	0·004
≥2 lifestyle risk factors	No	389	73	2·21 (1·60–3·06)	<0·0001
≥2 lifestyle risk factors	Yes	66	16	3·03 (1·76–5·20)	<0·0001

Lifestyle risk factors are current smoking, obesity, physical inactivity, and high alcohol consumption. HRs are adjusted for age and study. HR=hazard ratio.

## Discussion

Evidence from our pooling of individual-participant data from seven European cohort studies suggests that job strain is a risk factor for mortality in men with cardiometabolic disease, as defined by the presence of coronary heart disease, stroke, or diabetes. The mortality difference between groups with and without job strain was clinically significant and independent of socioeconomic status, the conventional and lifestyle risk factors measured (current smoking, obesity, physical inactivity, high alcohol consumption, hypertension, dyslipidaemia), and pharmacotherapy. This finding is unlikely to be attributable to type I error due to multiple testing because it was robust to correction for multiple comparisons. In absolute terms, the difference in age-standardised mortality among men with cardiometabolic diseases was greater between current and non-smokers than between men with and without job strain. However, high cholesterol, obesity, high alcohol consumption, and physical inactivity were associated with smaller mortality differences than job strain.

Our findings agree with those of a study of patients with acute myocardial infarction from the USA, in which individuals who reported life stress had higher mortality than those free of life stress (HR 1·4, 95% CI 1·2–1·8)^14^ and the few previous, small-scale prognostic studies on patients with cardiovascular disease,^23^, ^24^, ^25^, ^26^ which in combination suggest a 1·6 times (95% 1·2–2·2) increased risk of recurrent events associated with job strain.^17^ The observed associations are also biologically plausible. The stress hormone cortisol stimulates glucose production in the liver and antagonises the action of insulin in peripheral tissues—both processes have the potential to contribute to worse prognoses in people with diabetes.^5^, ^6^ Stress can also have adverse effects on cardiometabolic systems by inducing transient endothelial dysfunction, myocardial ischaemia, and cardiac arrhythmia and thus increasing the risk of fatal and non-fatal cardiac events.^6^

To our knowledge, this is the first large-scale study to examine the work stress–mortality association stratified by cardiometabolic risk profile. Our data showed that job strain substantially increased mortality risk even in subgroups of men with prevalent cardiometabolic disease but a favourable cardiometabolic risk profile, suggesting that standard care targeting conventional and lifestyle risk factors (eg, blood pressure, lipids, smoking, obesity, physical inactivity) does not necessarily mitigate the excess mortality risk associated with job strain. The European prevention guidelines^7^ and the American Heart Association policy statements^8^ highlight psychosocial stress as a potential barrier to healthy lifestyles and optimal medication adherence, and recommend management of stress in individuals with high cardiovascular risk or established cardiovascular disease. Our findings are consistent with these recommendations, but also suggest that harmful effects of stress in men were not attributable to the lifestyle risk factors measured or poor adherence to pharmacotherapy; excess mortality risk was observed even among patients successfully treated for cardiometabolic disease who were normotensive, non-obese, physically active, had normal blood cholesterol, and were not smokers or heavy drinkers.

There are various ways of expanding standard care to address work stress in patients, including systematic screening for stress and, if needed, interventions such as consultation, rehabilitation, job redesign, reductions in working hours, and retirement on health grounds.^6^, ^7^ In a Cochrane review of 35 randomised controlled trials including a total of 10 703 patients with coronary heart disease who had at least 6 months' follow-up, psychological interventions that alleviated stress and other psychological symptoms were successful in reducing cardiac mortality for people with coronary heart disease.^27^ However, it is unclear whether those interventions would benefit men with job strain and cardiometabolic disease.

For other groups stress-related differences in mortality were small or absent, both in relative and absolute terms. In working-aged women with cardiometabolic disease, for example, job strain was not associated with a significant increase in mortality risk and the absolute mortality difference between those with and without job strain was only 10·8 per 10 000 person-years (for comparison, the corresponding mortality difference in men was 52·1 per 10 000 person-years). Similarly, effort–reward imbalance was not associated with increased mortality in men or women with cardiometabolic disease, suggesting that job strain and effort–reward imbalance are of different prognostic value. Job strain encompasses only external sources of stress, whereas effort–reward imbalance also involves the individual's own behaviours. People with more severe cardiometabolic disease tend to shorten their working hours as a consequence of their condition, thus potentially reducing any effort–reward imbalance through reduced effort.^10^, ^28^ This change could mitigate the link between effort–reward imbalance and mortality. By contrast, external characteristics of work that relate to job strain remain unchanged after the onset of disease. Finally, as expected, in healthy people work stress did not substantially increase mortality risk, although in men free of cardiometabolic disease, we observed a moderate association between effort–reward imbalance and risk of death.

Our study benefits from a large sample size, predefined exposure assessment, coverage of several European countries, and a mortality outcome assessed via record linkage with very little loss to follow-up. The limitations of our study include the use of a single measurement of work stressors and risk factors, which does not include any measure of chronicity or change over time. There is also the possibility that prevalent cardiometabolic disease was underestimated in those studies with no measures of undiagnosed diabetes and cardiovascular disease (eg, silent myocardial infarctions). These drawbacks could contribute to an underestimation or overestimation of associations with mortality. We adjusted the associations for several conventional and lifestyle risk factors, but data for blood pressure and blood cholesterol concentration were not available in all the studies. This limitation could lead to overestimation of the status of job strain as an independent predictor of mortality, although there was no evidence to support this possibility in supplementary analyses of the three cohort studies with relevant data. We did not have detailed data on the duration or severity of the cardiometabolic diseases. Several factors that are more common in individuals with stress that can precipitate a fatal cerebrovascular or cardiovascular event, or otherwise increase risk of premature death, were not covered by our baseline measurement. These include, for example, stress-induced ischaemia, cardiac arrhythmia, low-grade systemic inflammation, increased blood viscosity, platelet activation and increases in the levels of coagulation and fibrinolytic factors, short and long sleep durations and sleep disorders, and reduced self-care.^6^, ^29^, ^30^ Further research is needed to establish the role of such factors in the excess mortality risk seen in men with job strain and cardiometabolic disease and to examine mechanisms underlying the observed sex differences in the effects of job strain.

In conclusion, the results of this large pan-European study suggest that in men with cardiometabolic disease, the contribution of job strain to risk of death is clinically significant and independent of conventional risk factors and their treatment, as well as the lifestyle factors measured. Subsequent research should employ intervention designs to establish whether systematic screening and management of work stressors, such as job strain, would contribute to improved health outcomes in men with prevalent coronary heart disease, stroke, or diabetes.
