# Supplementary material for: Work stress and risk of death in men and women with and without cardiometabolic disease: a multicohort study
Source: Lancet Diabetes Endocrinol. 2018 Sep;6(9):705–13. doi: 10.1016/S2213-8587(18)30140-2 (PMC6105619; doi:10.1016/S2213-8587(18)30140-2)

THE LANCET
Diabetes & Endocrinology

Supplementary appendix

This appendix formed part of the original submission and has been peer reviewed. We post it as supplied by the authors.

Supplement to: Kivimäki M, Pentti J, Ferrie J E. Work stress and risk of death in men and women with and without cardiometabolic disease: a multicohort study. *Lancet Diabetes Endocrinol* 2018; published online June 5. http://dx.doi.org/10.1016/S2213-8587(18)30140-2.

APPENDICES

Contents

	Page
**Web appendix 1: Description of the participating cohort studies**	2
eTable 1. Characteristics of participants at baseline	4
eTable 2. Characteristics of participants with prevalent cardiometabolic disease at baseline	5
**Web appendix 2: Assessment of work stressors**	6
**Web appendix 3: Additional analyses on all-cause mortality**	7
eFigure 1. Random-effects meta-analysis of the association between effort-reward imbalance at work and risk of death in men free of cardiometabolic disease	7
eFigure 2. Age-adjusted mortality per 10,000 person-years by status of effort-reward imbalance, smoking, alcohol consumption, physical inactivity, and obesity in men without a history of cardiometabolic disease	7
eTable 3. Minimally- and multivariable-adjusted hazard ratios for the association between number of work stressors and mortality by baseline cardiometabolic disease	8
eFigure 3. Age-adjusted mortality per 10,000 person-years by sex, prevalent cardiometabolic disease and number of work stressors as indicated by job strain and effort-reward imbalance at work	9
eFigure 4. Random-effects meta-analysis of the association between job strain and risk of death in men with prevalent cardiometabolic disease	9
eFigure 5. Hazard ratios and 95% confidence intervals for the association between job strain and mortality in men with cardiometabolic disease and a favourable risk profile with additional adjustments	10
**Web appendix 4: Analysis of cause-specific mortality**	11
eTable 4. Minimally adjusted hazard ratios and 95% confidence interval for the association between job strain and cause-specific mortality in men with cardiometabolic disease	11
eFigure 6. Age-adjusted deaths per 10,000 person-years by cause and job strain in men with cardiometabolic disease	11

Web appendix 1: Description of the participating cohort studies

Details of the design and recruitment of the participants in the studies included in our analyses are presented below. Participants were eligible for the present analysis if they were in employment and had available data on workplace stress and mortality.

Finnish Public Sector study (FPS)

The Finnish Public Sector study is a prospective cohort study comprising the entire public sector personnel of 10 towns (municipalities) and 21 hospitals in the same geographical areas. Participants, who were recruited from employers' records in 2000-2002, were individuals who had been employed in the study organisations for at least six months prior to data collection (1). 48 592 individuals (9 337 men and 39 255 women aged 17 to 65) responded to the questionnaire. Of these, 47 448 had data on workplace stress, baseline cardiovascular disease, and mortality and were eligible for our meta-analyses. Ethical approval was obtained from the ethics committee of the Finnish Institute of Occupational Health.

Gazel

Gazel is a prospective cohort study of 20 625 employees (15 011 men and 5 614 women) of France's national gas and electricity company, Electricité de France-Gaz de France (EDF-GDF) (2, 3) . Since the study baseline in 1989, when the participants were aged 35–50 years, they have been posted an annual follow-up questionnaire to collect data on health, lifestyle, individual, familial, social, and occupational factors. Job strain was measured in Gazel in 1997, which we treated as a baseline year for our analyses. 11 448 individuals participated that year and 11 362 of them had data on workplace stress, baseline cardiovascular disease, and mortality and were eligible for our meta-analysis. The GAZEL study received approval from the national commission overseeing ethical data collection in France (Commission Nationale Informatique et Liberté).

Health and Social Support (HeSSup)

The Health and Social Support (HeSSup) study is a prospective cohort study of a stratified random sample of the Finnish population in the following four age groups: 20–24, 30–34, 40–44, and 50–54. The participants were identified from the Finnish population register and posted an invitation to participate, along with a baseline questionnaire, in 1998 (4). Workplace stress was measured in 1998 and of the 25 898 individuals who responded to the questionnaire, 15 534 were in employment and had data on workplace stress, baseline cardiovascular disease, and mortality and were thus eligible for our meta-analyses. The Turku University Central Hospital Ethics Committee approved the study.

Still Working

Still Working is an ongoing prospective cohort study. In 1986, the employees (n = 12 173) at all Finnish centres of operation of Enso Gutzeit (a forestry products manufacturer) were invited to participate in a questionnaire survey on demographic, psychosocial and health-related factors. (5, 6). At baseline, 9 282 individuals responded, and of these 9 165 had data on workplace stress, baseline cardiovascular disease, and mortality and were eligible for our pooled analyses. The study was approved by the ethics committee of the Finnish Institute of Occupational Health.

Whitehall II

The Whitehall II study is a prospective cohort study set up to investigate socioeconomic determinants of health. At study baseline in 1985-1988, 10 308 civil service employees (6 895 men and 3 413 women) aged 35-55 and working in 20 civil service departments in London were invited to participate

in the study (7). Data on workplace stress, baseline cardiovascular disease, and mortality were available for 8 786 men of the men and women who were eligible for our meta-analyses. The Whitehall II study protocol was approved by the University College London Medical School committee on the ethics of human research. Written informed consent was obtained at each data collection wave.

WOLF (Work, Lipids, and Fibrinogen) Stockholm and WOLF Norrland studies

The WOLF (Work, Lipids, and Fibrinogen) Stockholm study is a prospective cohort study of 5 698 people (3 239 men and 2 459 women) aged 19–70 and working in companies in Stockholm county (8). WOLF Norrland is a prospective cohort of 4 718 participants aged 19-65 working in companies in Jämtland and Västernorrland counties (9). At study baseline the participants underwent a clinical examination and completed a set of health questionnaires. For WOLF Stockholm, the baseline assessment was undertaken at 20 occupational health units between November 1992 and June 1995 and for WOLF Norrland at 13 occupational health service units in 1996-98. Data workplace stress, baseline cardiovascular disease, and mortality were available from 5 666 participants from WOLF Stockholm and 4 702 participants from WOLF Norland and were included in the present analysis. The Regional Research Ethics Board in Stockholm, and the ethics committee at Karolinska Institutet, Stockholm, Sweden approved the study.

References

1. Kivimäki M, Lawlor DA, Smith GD, Kouvonen A, Virtanen M, Elovainio M, et al. Socioeconomic position, co-occurrence of behavior-related risk factors, and coronary heart disease: the Finnish Public Sector Study. *Am J Public Health.* 2007, 2007;97:874-9.
2. Goldberg M, Leclerc A, Bonenfant S, Chastang JF, Schmaus A, Kaniewski N, et al. Cohort profile: the GAZEL Cohort Study. *Int J Epidemiol.* 2007;36:32-9.
3. Zins M, Leclerc A, Goldberg M. The French GAZEL Cohort Study: 20 years of epidemiological research. *Advances in Life Course Research.* 2009;14:135-46.
4. Korkeila K, Suominen S, Ahvenainen J, Ojanlatva A, Rautava P, Helenius H, et al. Non-response and related factors in a nation-wide health survey. *Eur J Epidemiol.* 2001;17:991-9.
5. Kalimo R, Toppinen S. Organizational well-being: ten years of research and development: in a forest industry corporation. In: Kompier M, Cooper C, editors. Preventing Stress, Improving Productivity: European Case Studies in the Workplace. London: Routledge; 1999. p. 52-85.
6. Väänänen A, Murray M, Koskinen A, Vahtera J, Kouvonen A, Kivimäki M. Engagement in cultural activities and cause-specific mortality: prospective cohort study. *Prev Med.* 2009;49:142-7.
7. Marmot MG, Davey Smith G, Stansfeld S, Patel C, North F, Head J, et al. Health inequalities among British civil servants: the Whitehall II study. *Lancet.* 1991;337:1387-93.
8. Peter R, Alfredsson L, Hammar N, Siegrist J, Theorell T, P. W. High effort, low reward, and cardiovascular risk factors in employed Swedish men and women: baseline results from the WOLF Study. *J Epidemiol Community Health.* 1998;52:540-7
9. Alfredsson L, Hammar N, Fransson E, de Faire U, Hallqvist J, Knutsson A, et al. Job strain and major risk factors for coronary heart disease among employed males and females in a Swedish study on work, lipids and fibrinogen. *Scand J Work Environ Health.* 2002;28:238-48.

eTable 1. **Characteristics of participants at baseline**

	Population free of prevalent cardiometabolic disease (N = 99,222)		Population with prevalent cardiometabolic disease at baseline (N = 3,441)	
**Baseline characteristic**	N	% or Mean (SD)	N	% or Mean (SD)
Sex				
Men	42,533	42.9	1,975	57.4
Women	56,689	57.1	1,466	42.6
Mean age, years	99,222	43.8 (9.4)	3,441	48.3 (7.7)
Coronary heart disease, %*				
No	99,222	100.0	2,972	86.4
Yes	0	0	469	13.6
Stroke, %*				
No	99,222	100.0	3,074	89.3
Yes	0	0	367	10.7
Diabetes, %*				
No	99,222	100	750	21.8
Yes	0	0	2,691	78.2
Socioeconomic status, %				
High	23,797	24.4	778	23.1
Intermediate	48,594	49.9	1,598	47.4
Low	24,982	25.7	990	29.4
Smoking status, %				
Never smoker	41,933	44.0	1,253	37.6
Ex-smoker	33,538	35.2	1,357	40.7
Current smoker	19,861	20.8	725	21.7
Alcohol consumption, %				
None	11,598	11.9	556	16.4
Moderate	75,364	77.4	2,389	70.6
Risky	10,385	10.7	437	12.9
Physical inactivity, %				
Yes	20,791	21.5	1,045	31.3
No	75,938	78.5	2,297	68.7
BMI, mean	88,956	25.0 (3.8)	3,243	27.1 (4.7)
BMI category, %				
<18.5 (underweight)	1,093	1.2	30	0.9
18.5-24.9 (normal weight)	49,106	55.2	1,143	35.3
25-29.9 (overweight)	30,101	33.8	1,309	40.4
30 or higher (obese)	8,656	9.7	761	23.5
High blood pressure, %†				
No	15185	83.9	653	72.2
Yes	2911	16.1	252	27.9
High cholesterol, %†				
No	10917	62.3	375	45.7
Yes	6617	37.7	445	54.3
High adherence to pharmacotherapy, %‡				
No	64766	90.7	789	40.8
Yes	6650	9.3	1145	59.2

* Of the participants, 408 had a history only of coronary heart disease, 335 only stroke, 2619 only diabetes, 47 both coronary heart disease and diabetes, 18 stroke and diabetes, 7 coronary heart disease and stroke, and 7 all three conditions.
† This measure was available from the Whitehall, WOLF-S and WOLF-N studies.
‡ This measure was available from the FPS, HeSSup, WOLF-S and WOLF-N studies.

***eTable 2*. Characteristics of participants with prevalent cardiometabolic disease at baseline**

	Men (N = 1,975)		Women (N = 1,466)			Men	Women
**Baseline characteristic**	N	% or Mean (SD)	N	% or Mean (SD)	P-value	Mortality per 10,000 py	
Death, %					<0.0001		
No	1668	84.5	1363	93.0			
Yes	307	15.5	103	7.0		103.7	55.6
Job strain, %					<0.0001		
No	1734	87.8	1147	78.2		97.7	53.2
Yes	241	12.2	319	21.8		149.8	64.0
Mean age, years	1975	49.1 (7.1)	1466	47.3 (8.3)	<0.0001		
Coronary heart disease, %					<0.0001		
No	1579	80.0	1393	95.0		97.7	52.9
Yes	396	20.0	73	5.0		127.1	98.3
Stroke, %					0.71		
No	1761	89.2	1313	89.6		104.7	57.9
Yes	214	10.8	153	10.4		96.5	35.7
Diabetes, %					<0.0001		
No	550	27.9	200	13.6		107.7	54.6
Yes	1425	72.1	1266	86.4		102.3	55.8
Socioeconomic status, %					<0.0001		
Low	548	28.5	442	30.6		133.1	71.2
Intermediate	836	43.5	762	52.8		101.2	51.9
High	539	28.0	239	16.6		78.7	33.4
Smoking status, %					<0.0001		
Never smoker	587	30.5	666	47.3		89.4	40.3
Ex-smoker	906	47.0	451	32.0		83.5	42.1
Current smoker	433	22.5	292	20.7		164.0	102.0
Alcohol consumption, %					<0.0001		
None	220	11.4	336	23.3		135.3	52.6
Moderate	1381	71.2	1008	69.9		94.1	55.3
Risky	338	17.4	99	6.9		114.8	59.8
Physical inactivity, %					0.96		
Yes	599	31.2	446	31.3		105.9	75.5
No	1319	68.8	978	68.7		100.0	45.2
BMI, mean	1836	27.0 (4.0)	1407	27.3 (5.4)	0.04		
BMI category, %					<0.0001		
<18.5 (underweight)	7	0.4	23	1.6		192.4	190.5
18.5-24.9 (normal weight)	613	33.4	530	37.7		105.7	51.9
25-29.9 (overweight)	851	46.4	458	32.6		74.4	56.2
30 or higher (obese)	365	19.9	396	28.1		109.3	46.9
Lifestyle risks, %					0.75		
0	798	40.4	610	41.7		85.4	40.0
1	721	36.5	522	35.7		104.9	56.3
2-	455	23.1	331	22.6		135.5	80.6
High blood pressure*, %					0.0007		
No	438	68.9	215	79.9		105.4	83.1
Yes	198	31.1	54	20.1		120.7	68.5
High cholesterol *, %					0.23		
No	278	47.0	97	42.4		80.9	82.6
Yes	313	53.0	132	57.6		124.9	78.9
High adherence to pharmacotherapy†, %					<0.0001		
No	276	32.9	513	46.8		84.4	35.3
Yes	562	67.1	583	53.2		97.3	56.9

* This measure was available only from the Whitehall, WOLF-S and WOLF-N studies.
† This measure was available only from the FPS, HeSSup, WOLF-S and WOLF-N studies.

Web appendix 2: Assessment of work stressors

Reports from the IPD-Work consortium are based on pre-defined, harmonised and validated definitions of work stress.[1-3] Here we investigated two common work stressors, job strain and effort-reward imbalance at work.

Job strain was measured using sets of questions from the validated Job Content Questionnaire (JCQ) and Demand-Control Questionnaire (DCQ), included in the baseline self-report questionnaire of all of the studies.[2] For each participant, mean response scores were calculated for job demands (i.e., from questions about whether the participant had to work very hard, had an excessive amount of work, conflicting demands and not enough time) and job control items (from questions about decision freedom and learning new things at work). The Pearson correlations between the harmonised scales used in this study and complete versions of the Job Content and Demand Control Questionnaires were $r>0.9$ except for one study in which the correlation was $r=0.8$. According to the original and most commonly used categorisations, high job demands were defined as having a job demand score that was higher than the study-specific median score, and low job control a score that was lower. The exposure was defined as job strain (high demands and low control) versus no strain (all other combinations) according to the job strain model.[4]

The Effort-Reward Imbalance questionnaire was constructed in all the studies from questions regarding psychosocial aspects of the job. For each participant, mean response scores were calculated for effort (i.e., from questions about work demands and efforts) and reward items (from questions about monetary and non-monetary rewards at work). Pearson correlation coefficients between the harmonized scales used in this study and complete versions of the Effort-Reward Imbalance questionnaire were high; $r>0.9$ for the effort scales and $r>0.8$ for the reward scales.[3] Scores for effort-reward imbalance were calculated by dividing the harmonized reward scale by the harmonized effort scale. [3] Values >1 indicate that effort exceeds reward (an effort-reward imbalance). The exposure was defined as effort-reward imbalance vs. no effort-reward imbalance.[3]

To examine the combined effects of job strain and effort-reward imbalance, we constructed a 3-level exposure variable: 0=neither effort-reward imbalance nor job strain; 1=either job strain or effort-reward imbalance, but not both; and 2=both job strain and effort-reward imbalance.[5]

References

1. Kivimaki M, Singh-Manoux A, Ferrie JE, Batty GD. Post hoc decision-making in observational epidemiology--is there need for better research standards? *Int J Epidemiol*. 2013; 42: 367-70.
2. Fransson EI, Nyberg ST, Heikkila K, et al. Comparison of alternative versions of the job demand-control scales in 17 European cohort studies: the IPD-Work consortium. *BMC Public Health*. 2012; 12: 62.
3. Siegrist J, Dragano N, Nyberg ST, et al. Validating abbreviated measures of effort-reward imbalance at work in European cohort studies: the IPD-Work consortium. *Int Arch Occup Environ Health*. 2013; 87: 249-56.
4. Kivimaki M, Nyberg ST, Batty GD, et al. Job strain as a risk factor for coronary heart disease: a collaborative meta-analysis of individual participant data. *Lancet*. 2012; 380: 1491-7.
5. Dragano N, Siegrist J, Nyberg ST, et al. Effort-reward imbalance at work and incident coronary heart disease: a multi-cohort study of 90,164 individuals. *Epidemiol*. 2017; 28: 619-26.

Web appendix 3: Additional analyses on all-cause mortality

eFigure 1. **Random-effects meta-analysis of the association between effort-reward imbalance at work and risk of death in men free of cardiometabolic disease**

Study	N(Cases)	N(Total)	Hazard Ratio (95% CI)
WOLF-N	107	3708	0.87 (0.45-1.67)
WOLF-S	120	3066	1.55 (0.93-2.56)
FPS	265	8626	1.11 (0.86-1.43)
Gazel	346	6629	1.41 (1.07-1.85)
Whitehall II	257	5557	1.14 (0.89-1.47)
**Random effects model**			**1.21 (1.05-1.39)**

Heterogeneity: I-squared=0%, p=0.444

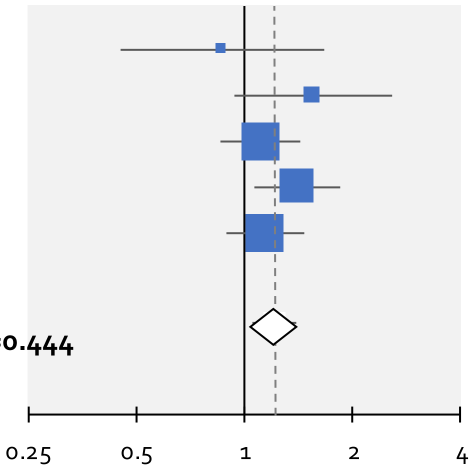

eFigure 2. **Age-adjusted mortality per 10,000 person-years by status of effort-reward imbalance, smoking, alcohol consumption, physical inactivity, and obesity in men without a history of cardiometabolic disease**

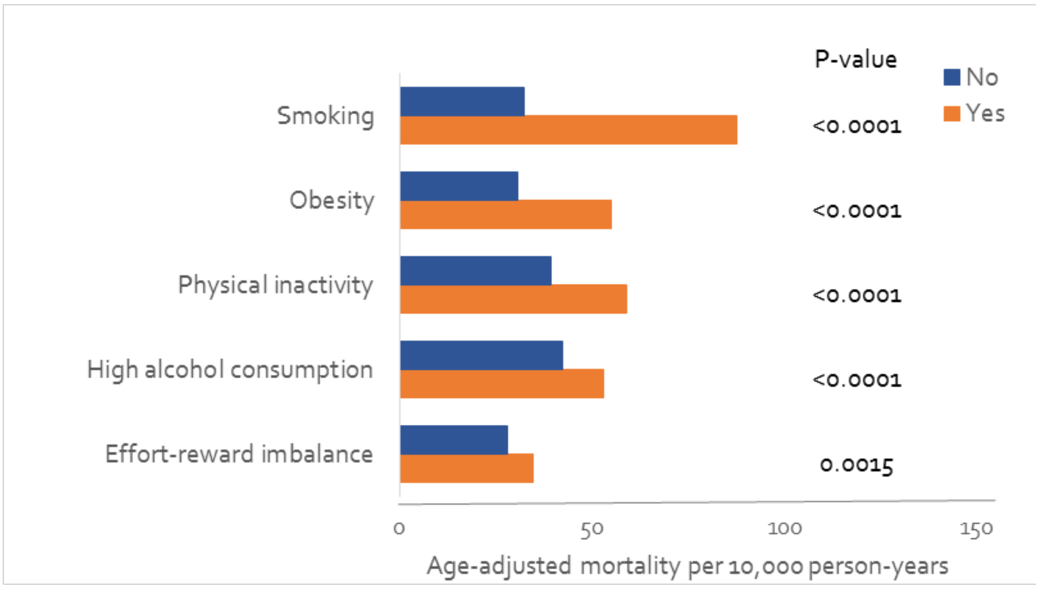

***eTable 3*. Minimally- and multivariable-adjusted hazard ratios (HR) for the association between number of work stressors and mortality by baseline cardiometabolic disease**

Work stress exposure	Population free of prevalent cardiometabolic disease (N = 72,477)					Population with prevalent cardiometabolic disease at baseline (N = 2,765)				
	N(death)/ N(total)	Age-adjusted HR (95% CI)	P	Multivariable adjusted* HR (95% CI)	P	N(death)/ N(total)	Age-adjusted HR (95% CI)	P	Multivariable adjusted* HR (95% CI)	P
**Men**										
Number of stressors			0.04		0.08			0.02		0.19
0	703/18,298	1.00 (reference)		1.00 (reference)		120/961	1.00 (reference)		1.00 (reference)	
1	316/7,510	1.15 (1.00-1.33)		1.16 (1.01-1.34)		51/444	0.72 (0.51-1.01)		0.78 (0.55-1.10)	
2	76/1,778	1.26 (0.99-1.61)		1.18 (0.92-1.51)		23/120	1.40 (0.89-2.22)		1.22 (0.74-2.00)	
**Women**										
Number of stressors			0.14		0.26			0.42		0.43
0	508/26,059	1.00 (reference)		1.00 (reference)		42/643	1.00 (reference)		1.00 (reference)	
1	215/13,080	0.88 (0.75-1.03)		0.87 (0.74-1.03)		21/395	0.83 (0.49-1.41)		0.80 (0.46-1.39)	
2	120/5,752	1.08 (0.89-1.32)		1.01 (0.82-1.24)		15/202	1.30 (0.72-2.35)		1.25 (0.68-2.32)	

*Minimally adjusted models include age and study as covariates. Multivariable adjusted models include age, study, smoking, physical inactivity, alcohol consumption, BMI, and socioeconomic status as covariates.

eFigure 3. **Age-adjusted mortality per 10,000 person-years by sex, prevalent cardiometabolic disease and number of work stressors as indicated by job strain and effort-reward imbalance at work**

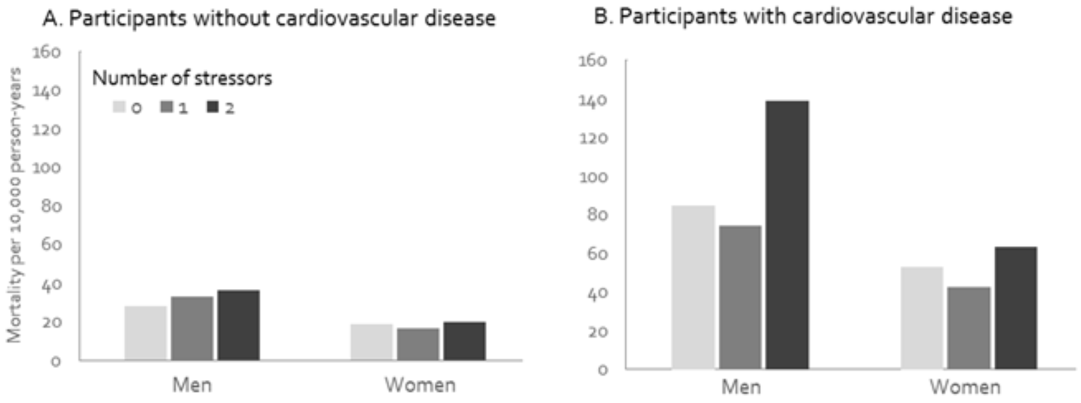

eFigure 4. **Random-effects meta-analysis of the association between job strain and risk of death in men with prevalent cardiometabolic disease**

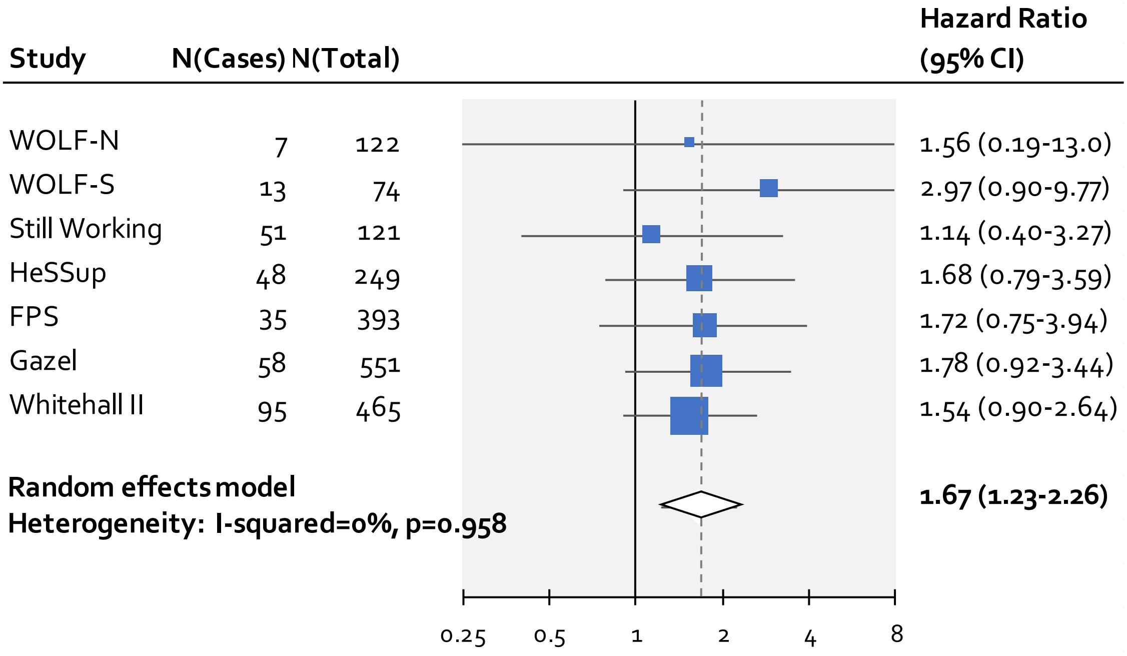

***eFigure 5.* Hazard ratios and 95% confidence intervals for the association between job strain and mortality in men with cardiometabolic disease and a favourable risk profile with additional adjustments.**

Analysis of the subgroup of participants with no lifestyle risk factors is adjusted for age and study. In analyses of subgroups of normotensive participants, those with no dyslipidaemia, normotensive participants with no dyslipidaemia, and participants with high adherence to pharmacotherapy, systolic blood pressure and total cholesterol, both treated as continuous variables, were added as covariates

Healthy subgroup	Hazard ratio (95% CI)	P-value	N (total)	N (deaths)
No lifestyle risk factors				
No job strain	1.00		718	88
Job strain	2.01 (1.18 - 3.43)	0.0100	80	16
Normotensive (syst/diast blood pressure <140/90 mmHg)				
No job strain	1.00		372	54
Job strain	3.77 (1.92-7.39)	0.0001	66	20
No dyslipidemia (total cholesterol <6.2 mmol/l)*				
No job strain	1.00		241	26
Job strain	2.26 (0.89-5.72)	0.0860	37	8
Normotensive and no dyslipidemia				
No job strain	1.00		145	10
Job strain	6.17 (1.74-21.9)	0.0049	30	8
High adherence to pharmacotherapy†				
No job strain	1.00		498	58
Job strain	2.38 (1.34 - 4.20)	0.0029	64	15

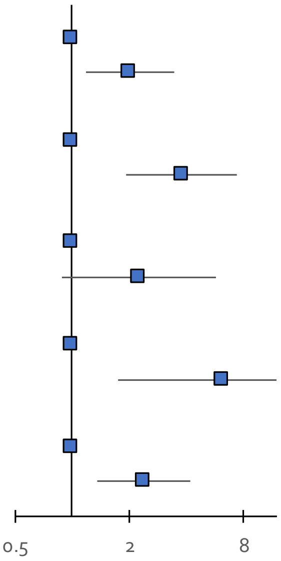

* For a subgroup of participants with total cholesterol <5.0 mmol/l, the corresponding hazard ratio is 7.72 (95% CI 1.23-48.5) for those with job strain (N=9, 3 deaths) compared to those without job strain (N=71, 5 deaths).

† Antidiabetic (ATC A10), antihypertensive (ATC C02, C03, C07-C09), lipid-lowering (ATC C10AA), and anticoagulation (ATC B01) medication.

Web appendix 4: Analysis of cause-specific mortality

We used codes from the International Classification of Diseases, 10th Revision (ICD-10) to define two largest causes of death, cardiovascular disease (I00–I99) and cancer (C00–C97). Other causes of death included all remaining noncancer, noncardiovascular disease deaths. In statistical analyses of cause-specific mortality, we used Cox regression and examined separately associations of work stressors with cardiovascular disease mortality, cancer mortality, and noncardiovascular, noncancer mortality. Results are reported in etable 4 and efigure 5 and are summarised in article result section.

***eTable 4*. Minimally adjusted hazard ratios and 95% confidence interval for the association between job strain and cause-specific mortality in men with cardiometabolic disease**

Cause of death	N (total)	N (deaths)	Hazard ratio (95% CI)	P-value
Cardiovascular disease				
No job strain	1734	115	1.00 (reference)	
Job strain	241	22	1.71 (1.08 - 2.71)	0.02
Cancer				
No job strain	1734	63	1.00 (reference)	
Job strain	241	13	1.75 (0.96 - 3.19)	0.07
Non-cardiovascular, non-cancer				
No job strain	1734	63	1.00 (reference)	
Job strain	241	11	1.33 (0.70 - 2.53)	0.38

*Minimally adjusted models include age and study as covariates.

***eFigure 6*. Age-adjusted deaths per 10,000 person-years by cause and job strain in men with cardiometabolic disease.** CVD, cardiovascular disease.

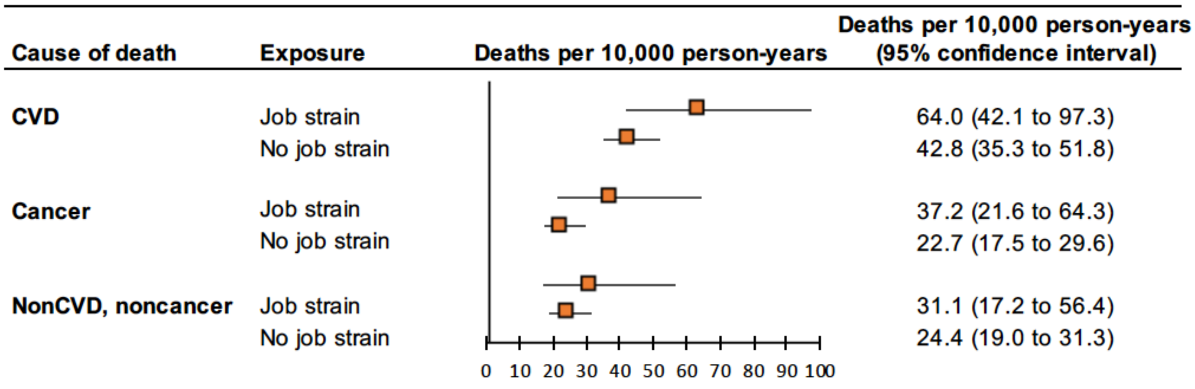

Supplement: Supplementary appendix [file mmc1.pdf]
